# Increasing Patient Safety Among Multiple Sclerosis (MS) Drug Dalfampridine Users by Expanding Awareness of the Serious Side Effects

**DOI:** 10.7759/cureus.20884

**Published:** 2022-01-03

**Authors:** Rameez Zaman, Elliott Goldberg, Naseem Zomorodi, Vinita J Acharya

**Affiliations:** 1 Neurology, Penn State College of Medicine, Hershey, USA; 2 Neurology/Epilepsy, Penn State College of Medicine, Hershey, USA

**Keywords:** dalfampridine, ms, multiple sclerosis, ampyra, seizure, risk of seizure, ms drug, faers

## Abstract

Dalfampridine is a drug used to improve walking in multiple sclerosis (MS) patients. Approved in 2010, it is contraindicated in patients who have a history of seizures and/or renal disease. In this case report, we present a patient who did not have either of these contraindications yet had a seizure episode with the only contributing factor being the use of dalfampridine.

## Introduction

Multiple sclerosis (MS) often reduces patients' functional abilities, with 90% of MS patients reporting difficulty in walking [1]. Dalfampridine, a potassium channel inhibitor approved by the FDA in 2010, remains the only approved medication to improve gait disturbance in MS patients. It is thought to work by prolonging action potentials in demyelinated neurons [2]. Dalfampridine is contraindicated in patients who have had a history of prior seizures and have a creatinine clearance less than or equal to 50 mL/min. In addition, the FDA recommends caution in the use of dalfampridine in patients with a creatinine clearance between 51 and 80 mL/min [3]. Below we describe an otherwise healthy MS patient, with no history of seizures and a creatinine clearance above 80 mL/min, who presented with a seizure episode. The only potentiating factor for seizures, in this case, was the patient’s use of dalfampridine (other etiologies such as trauma, tumor, stroke, and infectious causes were ruled out in the emergency department [ED]). Seizures in otherwise healthy MS patients who are using dalfampridine have not been readily documented.

## Case presentation

**Figure 1:**
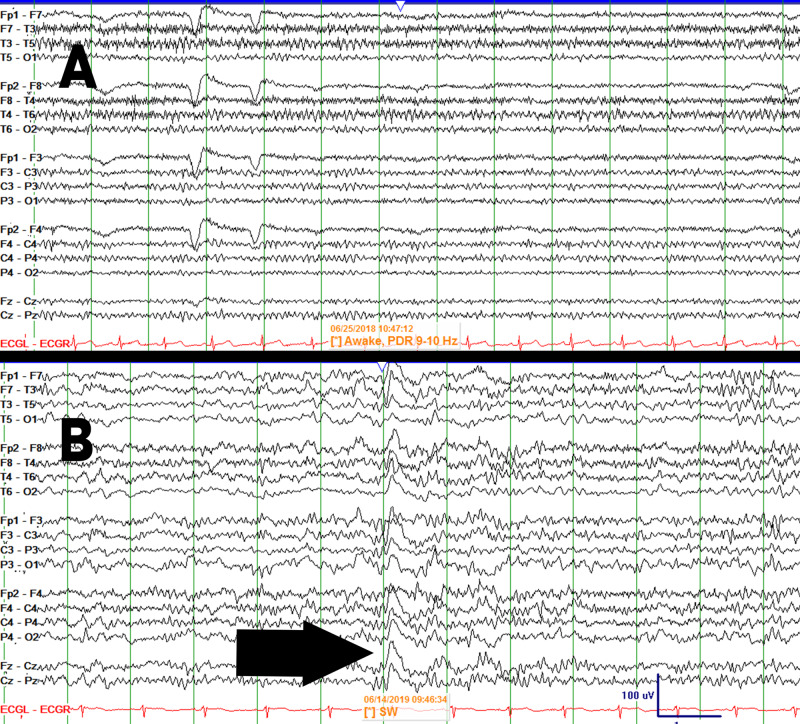
Routine EEG of the patient in 2018 and 2019 (A)  The patient's 2018 EEG showing normal background activity, ruling out a seizure. (B) The patient's 2019 EEG showing a left frontal sharp wave (black arrow) indicating epileptic activity, confirming seizure. No focus of seizure was noted in the right parietal cortex.

**Figure 2:**
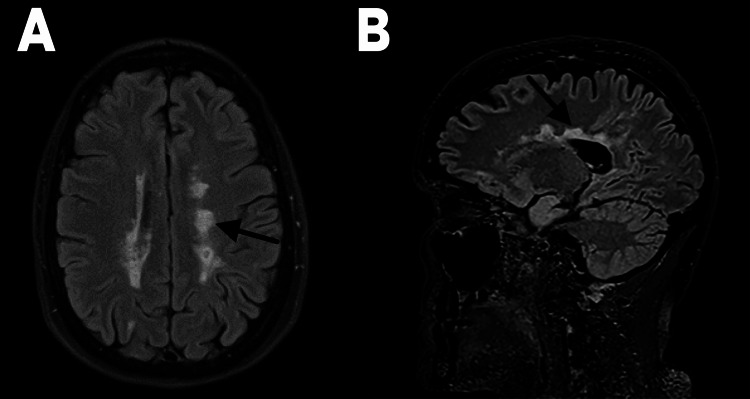
(A, B) FLAIR MRI performed during 2019 seizure admission for seizure activity showing periventricular hyperintensities (black arrows) consistent with her prior 2018 MRI images.

A 61-year-old right-handed Caucasian woman was diagnosed with MS in 1987 with a relapsing-remitting course. In 1993, she was started on Interferon beta-1b. In 2010, after endorsing significant leg weakness, unsteadiness, and decreased endurance, she was started on dalfampridine, which improved her walking ability. In 2018, she reported to the ED for a seizure-like event. Although seizure activity was ruled out clinically by history and EEG with no epileptiform activity (Figure 1A), it was recommended she discontinue the dalfampridine. The patient asked to continue on dalfampridine due to her marked improvement in ambulation with the medication. After further consideration of her quality of life, and without predisposing risk factors to seizures, preexisting renal disease, or definitive evidence of seizures, her neurologist again prescribed dalfampridine. Approximately nine months prior to this event, she began a trial of peginterferon beta-1a to replace the interferon beta-1b. The patient expressed that the peginterferon beta-1a was not working and felt that it may have been the offending agent for the episode and asked to be switched to a new medication. In July 2018, she was started on dimethyl fumarate. She then reported to the ED in 2019 for another seizure event. This time history and workup confirmed the diagnosis of seizure. The routine EEG confirmed epileptiform activity in the left frontal region (Figure 1B). An MRI was also done at the time (Figures 2A, 2B), revealing no changes in her demyelinating plaque distribution when compared to an MRI from March 2018. She was started on levetiracetam 750 mg twice daily and her dalfampridine was discontinued for risk of future seizures. Later, ambulatory EEG showed no abnormalities, focal slowing, or epileptiform activity.

## Discussion

**Figure 3:**
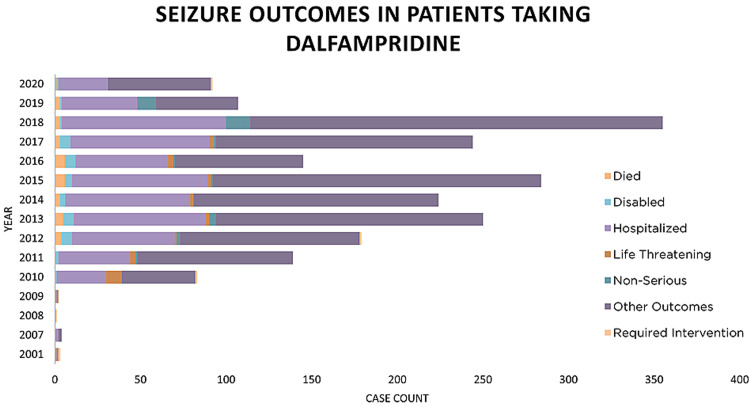
Reported outcomes of patients taking dalfampridine after having a seizure This graph, with data obtained from the FAERS Public Dashboard, displays reported outcomes of seizures in patients taking dalfampridine [4].

Although dalfampridine can significantly improve walking in people with MS, clinicians should consider the risk of seizures and must openly discuss it with patients. It is important to recognize that the seizures can occur in patients with no prior history of seizures. As can be seen in data obtained from the FDA Adverse Event Reporting System (FAERS) Public Dashboard (Figure 3), the outcomes of these seizures can be disabling, and even fatal, which is why these conversations should be commonplace when prescribing dalfampridine [4].

The seizure risk increase is directly correlated with elevated serum concentrations of dalfampridine. Greater than 90% of a dalfampridine dose is renally eliminated. Thus, patients with renal impairment have a heightened risk of seizures as a result of dalfampridine accumulating in the serum [5]. Dalfampridine should not be used in patients with a history of seizures or moderate to severe renal impairment as measured by creatinine clearance.

New-onset seizures in patients on dalfampridine should raise suspicion for a drug-induced reaction, and the medication must be discontinued. Although epileptiform activity was ruled out in the aforementioned patient's first seizure-like episode, immediate lifetime discontinuation of dalfampridine could have prevented her second episode, which was confirmed to be a seizure by EEG (Figure 1B). There are common medications, such as antipsychotics, which reduce the seizure threshold. However, in cases of seizures in patients taking antipsychotics or other potentially seizure-inducing drugs, the patient’s first seizure episode often leads to evaluation for seizures and prescription of antiepileptic medications with the continuation of the original antipsychotic medication. This is different in the case of dalfampridine.

The FDA made a safety announcement in 2012 regarding dalfampridine. Using information from the FAERS, several post-marketing case reports of seizures associated with dalfampridine at the labeled recommended dose were identified, with many cases occurring within the first week of starting the drug [4]. Moreover, the vast majority of seizures occurred in patients without a prior history of seizures [6].

## Conclusions

Considering these facts, the FDA’s recommendation in the case of dalfampridine, unlike other medications that may cause seizures, is to stop the medication permanently once there is a single seizure episode. There is a need to emphasize this fact and increase awareness of this serious side effect of dalfampridine among health care professionals to ensure patient safety. In addition, increased patient safety may be achieved by appropriately warning patients to stop the medication immediately if a seizure occurs and to contact their neurologist at once. This information could be extremely important for primary care and emergency medicine physicians who will likely be two healthcare providers that will initially manage a patient taking dalfampridine with a recent seizure.
